# Rationale and design of the acetazolamide as a chloride sparing diuretic in patients admitted with heart failure (ADA-HF) trial

**DOI:** 10.1093/ehjopen/oeaf019

**Published:** 2025-03-13

**Authors:** Joseph J Cuthbert, Elton Luo, Aia S M Ahmed, Meenakshy Ajith, Hafiz Butt, Henrietta Pinhol, Felix Baffour Korsah, Jeanne Bulemfu, Sarah Ford, Grant Constable, Leanne Cox, Alan S Rigby, Andrew L Clark

**Affiliations:** Clinical Sciences Centre, Hull York Medical School, University of Hull, Cottingham Road, Kingston-Upon-Hull HU6 7RX, UK; Department of Cardiology, Hull University Teaching Hospital NHS Trust, Castle Hill Hospital, Castle Road, Cottingham, Kingston-Upon-Hull HU16 5JQ, UK; Department of Cardiology, Hull University Teaching Hospital NHS Trust, Castle Hill Hospital, Castle Road, Cottingham, Kingston-Upon-Hull HU16 5JQ, UK; Department of Cardiology, Hull University Teaching Hospital NHS Trust, Castle Hill Hospital, Castle Road, Cottingham, Kingston-Upon-Hull HU16 5JQ, UK; Department of Cardiology, Hull University Teaching Hospital NHS Trust, Castle Hill Hospital, Castle Road, Cottingham, Kingston-Upon-Hull HU16 5JQ, UK; Department of Cardiology, Hull University Teaching Hospital NHS Trust, Castle Hill Hospital, Castle Road, Cottingham, Kingston-Upon-Hull HU16 5JQ, UK; Department of Cardiology, Hull University Teaching Hospital NHS Trust, Castle Hill Hospital, Castle Road, Cottingham, Kingston-Upon-Hull HU16 5JQ, UK; Department of Cardiology, Hull University Teaching Hospital NHS Trust, Castle Hill Hospital, Castle Road, Cottingham, Kingston-Upon-Hull HU16 5JQ, UK; Department of Cardiology, Hull University Teaching Hospital NHS Trust, Castle Hill Hospital, Castle Road, Cottingham, Kingston-Upon-Hull HU16 5JQ, UK; Department of Cardiology, Hull University Teaching Hospital NHS Trust, Castle Hill Hospital, Castle Road, Cottingham, Kingston-Upon-Hull HU16 5JQ, UK; Research and Development, Hull University Teaching Hospital NHS Trust, Castle Hill Hospital, Castle Road, Cottingham, Kingston-Upon-Hull HU16 5JQ, UK; Research and Development, Hull University Teaching Hospital NHS Trust, Castle Hill Hospital, Castle Road, Cottingham, Kingston-Upon-Hull HU16 5JQ, UK; Hull York Medical School, University of Hull, Cottingham Road, Kingston-Upon-Hull HU6 7RX, UK; Department of Cardiology, Hull University Teaching Hospital NHS Trust, Castle Hill Hospital, Castle Road, Cottingham, Kingston-Upon-Hull HU16 5JQ, UK

**Keywords:** Acetazolamide, Diuretic, Heart failure, Chloride

## Abstract

**Aims:**

The Acetazolamide as a chloride-sparing Diuretic in patients Admitted with Heart Failure (ADA-HF) trial will assess the safety and diuretic effect of oral ACZ given alongside a high-dose IV loop diuretic in patients admitted to the hospital with heart failure (HF) and severe fluid retention. Hypochloraemia is common in patients with HF and is associated with worse outcomes, but there are few treatment options available: we will also assess whether ACZ reduces urine chloride loss.

**Methods and results:**

The ADA-HF trial is a single centre, open-label, randomized-controlled trial of ACZ 250 mg twice daily plus standard care vs. standard care alone. The trial duration is 4 days. We will recruit 50 patients with severe peripheral oedema due to HF requiring standard care (240 mg of IV furosemide per day given via continuous infusion at 10 mg per hour). The co-primary endpoints are (1) the difference in net fluid loss daily, and over 4 days; and (2) difference in serum chloride concentrations between baseline and day 4. The trial has 80% power to detect a difference in fluid balance of 500–1000 mL per day; and a difference in serum chloride concentration of 1 mmol/L per day. Secondary endpoints include but are not limited to: time to recruit per patient; rate of adverse events; rate of recruitment; and cause-specific rate of drop-out of the study.

**Conclusion:**

ACZ may be a useful adjunct to diuretic therapy, but the safety and diuretic efficacy of oral ACZ when used alongside high-dose loop diuretics is unknown. ADA-HF will complement the ADVOR trial and may clarify what role ACZ may have for patients with severe congestion.

**Trial registration:**

ISRCTN registry. ISRCTN13060336. Registered on 09/02/2023. URL: https://doi.org/10.1186/ISRCTN13060336.

## Introduction

Severe fluid retention is the most common reason for hospitalisation in patients with heart failure (HF),^1^ and is associated with disability, poor quality of life, and an adverse prognosis.^2^ The standard treatment for fluid retention in patients admitted to hospital with HF is intravenous (IV) loop diuretic.^3^ Combining loop diuretics with another diuretic agent may increase diuresis, but may also increase the risk of diuretic side effects such as hypochloraemia.^4^

Hypochloraemia is a common side effect of high-dose loop diuretics, which is associated with adverse outcome.^5–7^ Whether hypochloraemia is a marker or mediator of poor prognosis is unknown, but it is a potential target for treatment.^8^ There are few ways of increasing serum chloride, but dietary supplements require enormous doses to affect only modest changes,^9^ and there is no data on IV supplementation from trials of hypertonic saline.^10^

**Figure 1:**
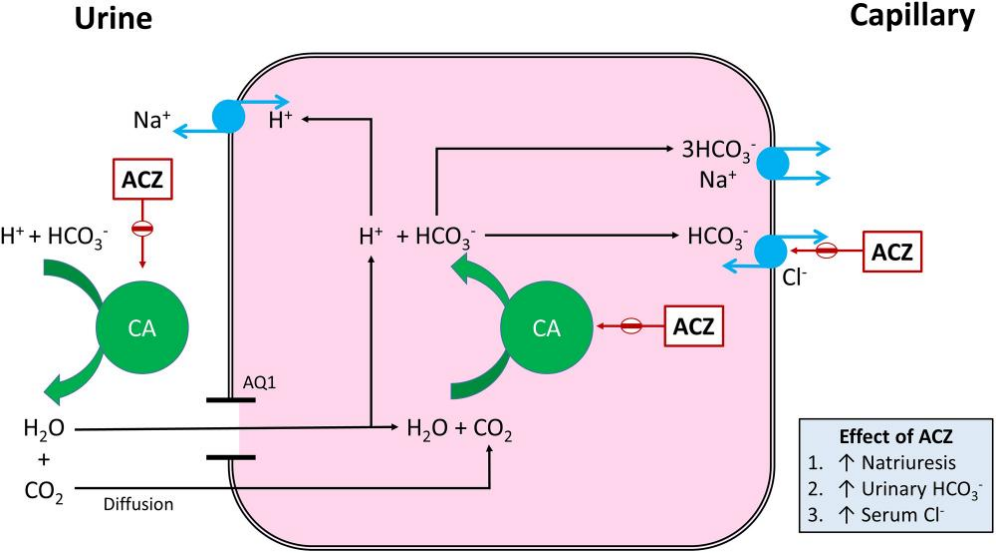
Mechanism of action of Acetazolamide. ACZ, Acetazolamide.

Acetazolamide is a carbonic anhydrase (CA) inhibitor. CA catalyses the interconversion between carbon dioxide and water on the one hand, and hydrogen (H^+^) and bicarbonate ions on the other. Acetazolamide increases chloride reabsorption from the urine *in vivo*,^11^ and increases serum chloride in humans.^12^ There are two potential mechanisms: firstly, increased ${\mathrm{HCO}}_{3}^{-}$ increases the negative charge in the urinary space, thus increasing the electrochemical gradient along which chloride is reabsorbed in the proximal convoluted tubule (PCT). Secondly, *in vivo* studies suggest that ACZ, separately from CA inhibition, also inhibits the basolateral $Cl^{-}{/\mathrm{HCO}}_{3}^{-}$ antiporter in the PCT thus reducing movement of chloride out of the blood and into the cell (*Figure 1*).^8^ Whether acetazolamide acts as a ‘chloride sparing’ agent to correct or prevent hypochloraemia during treatment with high-dose loop diuretics has never been tested in a randomized controlled trial (RCT).

As well as increasing renal chloride reabsorption, acetazolamide also increases diuresis. The majority of renal sodium reabsorption (∼70%) occurs in the PCT,^13^ via the sodium-hydrogen (Na^+^/H^+^) antiporter.^14^ Increased intracellular H^+^ concentration increases the activity of the Na^+^/H^+^ antiporter, which promotes sodium reabsorption from the urinary space.^15^ Inhibition of CA reduces sodium reabsorption in the proximal tubule, causing a natriuresis. The efficacy of loop diuretics is dependent, in part, on the amount of sodium that is delivered to the loop of Henle and distal convoluted tubule.^16^ Increasing natriuresis in the PCT may increase the efficacy of loop diuretics as well as induce a diuresis itself.

All trials of acetazolamide in patients admitted with HF to date have used low doses of IV loop diuretic as a comparator, limiting the generalisability of the data (*Table 1*).^17–19^ The largest trial, ADVOR, found that IV acetazolamide given alongside IV loop diuretic to patients admitted with HF caused a greater diuresis and was associated with greater rates of decongestion compared with IV loop diuretic alone, However, the median dose of furosemide was only 120 mg per day, and treatment was only given for 3 days. It is unclear what, if any, diuretic effect acetazolamide has when given alongside *high* doses of loop diuretic, nor what effect acetazolamide may have on serum chloride levels during treatment with loop diuretics. We designed the Acetazolamide as a chloride-sparing diuretic in patients admitted with HF (ADA-HF) trial to assess the safety and efficacy of oral acetazolamide given alongside high-dose IV loop diuretic in patients admitted to the hospital with HF and severe fluid retention.

**Table 1:** Randomized trials of Acetazolamide in patients admitted to hospital with heart failure

Trial (date)	*n*	ACZ dose and duration	Comparator	Findings
Imiela et al.^18^	20	<75 kg – 250 mg PO OD	Standard care.	Greater fluid loss (negative fluid balance) in ACZ arm between days 3–4
		75–100 kg – 375 mg PO OD	Mean IV furosemide dose 104 mg per day in ACZ arm vs. 136 mg per day in SoC arm	
		>100 kg–500 mg PO OD		
		Duration: 5 days		
DIURESIS-CHF^17^	34	250 mg OD^a^	Bumetanide at 2 × pre-admission dose.	No difference in natriuresis after 24 h of treatment.
		Duration: 3 days	Mean furosemide equivalent dose 135 mg per day in ACZ arm vs. 240 mg per day in the control arm^a^	
ADVOR^19^	519	500 mg OD IV	Placebo.	More decongestion in ACZ arm vs. placebo (42% vs. 30%; *P* < 0.001)
			Median IV furosemide	
		Duration: 3 days	dose in each arm 120 mg per day	Greater diuresis with ACZ vs. placebo (4.5L vs. 4.0L)

^a^Unclear if given PO or IV.

ACZ, acetazolamide; L, litres; mg, milligrams; *n*, number; OD, once daily; PO, per os; SoC, standard of care.

## The ADA-HF trial

### Study design

ADA-HF is an open-label, randomized-controlled trial in a single tertiary centre in Kingston-Upon-Hull (East Yorkshire, UK) that serves a population of ∼600 000 people. Consenting patients who are admitted to the hospital with a primary diagnosis of severe fluid retention due to HF will be randomized to oral acetazolamide 250 mg twice daily plus standard care or standard care alone. Recruitment began in March 2023 and ended in January 2024.

### Study hypothesis and objectives

The first main objective of the trial is to establish whether oral acetazolamide has an additive diuretic effect when given with high-dose IV furosemide. The second main objective is to establish whether acetazolamide can effect serum chloride concentration during treatment with high-dose IV loop diuretic. Our hypothesis is that inhibition of CA in the PCT will increase natriuresis, thus inducing a diuresis and enhancing the effect of loop diuretics, and will increase serum chloride reabsorption in the proximal tubule through two mechanisms (*Figure 1*).

Other study objectives include assessing the safety of oral acetazolamide in this patient population and the feasibility of performing a trial of diuretic interventions in patients admitted to hospital with HF in the UK.

### Endpoints

The co-primary endpoints are:

1. Difference in mean net fluid loss daily and over a 4-day period calculated as total volume intake in millilitres (mL)—total volume passed as urine in mL.
2. Difference in serum chloride concentrations between day 1 and day 4 calculated as serum chloride concentration on day 5 (mmol/L)—serum chloride concentration on day 1 (mmol/L).

Secondary and exploratory endpoints are shown in Box 1.

**Box 1:** Secondary and exploratory endpoints

**Secondary endpoints**
Change in body weight between day 1 and day 5
Change in serum sodium, bicarbonate and potassium concentrations between day 1 and day 5
Change in renal function measured by serum creatinine (µmol/L) and eGFR calculated using the Cockroft-Gault formula (mL/min/1.72 m^2^) between day 1 and day 5
Change in clinical assessment of congestion between day 1 and day 5
Change in IVC diameter between day 1 and day 5
Change in patient reported symptoms between day 1 and day 5 measured using a 5-point Likert scale
Difference in urinary electrolyte concentration 24–48 h and 72–96 h
Difference in the incidence of hyponatraemia (<135 mmol/L), hypochloraemia (<96 mmol/L), hypokalaemia (<3.5 mmol/L), high (>30 mmol/L) or low (<22 mmol/L) serum bicarbonate.
Difference in time to clinical euvolaemia and end of IV therapy
Difference in hospital length of stay
**Exploratory endpoints**
Rate of adverse reactions, serious adverse reactions and suspected unexpected serious adverse reactions in the treatment arm
Difference in the rate of adverse events and serious adverse events
Rate of recruitment to study
Time taken to screen and recruit a patient
Cause specific rate of drop-out after randomisation
Cause specific hospitalisation or mortality after 30 days and 180 days follow-up.

### Eligibility

All inclusion and exclusion criteria are shown in Box 2. Inclusion criteria have been kept broad purposefully to capture a representative population of patients admitted with HF. Patients with either a new or existing diagnosis of HF are eligible regardless of pre-admission loop diuretic treatment as long as severe fluid retention due to HF is deemed to be the primary reason for hospitalisation.

**Box 2:** Inclusion and exclusion criteria

**Inclusion criteria**
1) Aged >18 of any gender and able to give informed consent (females of child bearing age must consent to and have a negative pregnancy test prior to randomisation)
2) Heart failure of any aetiology (new or established diagnosis)
3) Admitted to hospital with a primary diagnosis of peripheral oedema caused by HF and deemed by treating clinicians to require treatment with IV diuretic.
4) Deemed to require standard of care—10 mg/h continuous furosemide infusion.
5) Patients whose medications have been discontinued for other reasons ≥48 previously may be considered eligible. These medications include; SGLT2I, thiazide or thiazide-like diuretics, high dose aspirin (≥500 mg/day), methotrexate, lithium, Sando-K®, sodium bicarbonate, other sodium tablets, oral steroids, or sodium valproate.
**Exclusion criteria**
1) Unable to give informed written consent
2) Allergy or contraindication to CA inhibitors
3) Patient thought to be at end-of-life
4) Concurrently taking thiazide (or thiazide-like) diuretic or sodium-glucose linked transporter-2 inhibitor, high dose aspirin (>500 mg/day), methotrexate, lithium, or sodium valproate, oral steroids Sando-K®, oral sodium bicarbonate, or other sodium tablets
5) Severe anaemia (haemoglobin ≤8 g/DL); or severe infection (investigator opinion).
6) SBP ≤80 mmHg at randomisation
7) Serum sodium (severe hyponatraemia) < 130 mmol/L at randomisation
8) Serum potassium (hypokalaemia) < 3.5 mmol/L at randomisation
9) Serum chloride (severe hyperchloraemia) > 110 mmol/L at randomisation
10) Severe renal dysfunction with an eGFR (estimate glomerular filtration rate) result of ≤20 mL/min calculated by the Cockcroft-Gault formula
11) Pregnant or intends to become pregnant whilst taking part in the trial

We have not specified a severity of peripheral oedema, nor an *n*-terminal pro-B-type natriuretic peptide (NTproBNP) cut off as most patients admitted to hospital in the UK have moderate to severe oedema (to the level of the knee at least),^1^ and all patients should have NTproBNP soon after admission to confirm or refute the diagnosis of HF—those with low levels will not have HF as the primary problem and are thus not eligible.^20^ Patients can be considered eligible for inclusion at any stage during their hospitalisation as long as they are deemed to require treatment with standard care.

Patients with severe electrolyte abnormalities, hypotension (systolic blood pressure ≤80 mmHg), or severe anaemia (serum haemoglobin <8 g/L), infection (treated with IV antibiotics), or renal dysfunction [estimated glomerular filtration rate (eGFR) measured using the Cockroft-Gault formula <30 mL/min/1.73m^2^] at screening will be excluded. Patients who were taking the following medications in 48 h prior to screening will also be excluded: high-dose aspirin (≥500 mg/day), lithium, methotrexate, or sodium valproate due to possible interactions with acetazolamide,^21^ and oral potassium, bicarbonate, or sodium salts due to confounding of the serum and urine electrolyte analysis.

We will also exclude patients taking either sodium glucose co-transporter 2 inhibitors (SGLT2I), thiazide or thiazide-like diuretics, or oral steroids due to confounding the diuretic effect of acetazolamide. This will ensure the only comparison in diuretic effect being made in the trial is between loop diuretic and loop diuretic plus oral acetazolamide. Mineralocorticoid receptor antagonists will be allowed as they do not have a diuretic effect at low doses.^22^

### Standard care and intervention

Acetazolamide will be given as oral tablets 250 mg twice daily to be taken 4–6 h apart for 4 days. The timeframe has been chosen as it is half the median length of hospitalisation of patients admitted with HF treated on cardiology wards in the UK.^2^ However, factors other than decongestion, such as social circumstances, may influence the timing of discharge, and 4 days was chosen as a timeframe during which most (if not all patients) will be requiring treatment with IV loop diuretics.

Patients in the acetazolamide arm will receive the first dose of acetazolamide the same day if randomized before 1200 noon, or the subsequent day at 0800 if randomized later. The continuous furosemide infusion will continue in the background for all patients. Patients randomized to the standard care arm will begin the trial at the time of randomisation.

According to current guidelines, the starting dose of IV furosemide for patients admitted to the hospital could range anywhere between 20 and 160 mg per day.^4^

The Diuretic Optimisation Strategies Evaluation (DOSE) trial found greater diuresis with high dose (2.5 × usual dose—median daily dose 258 mg) than with low dose (1 × usual dose—median daily dose 119 mg) loop diuretic.^23^ Most patients admitted with HF in the UK require high-dose loop diuretic treatment: if lower doses were appropriate, then it is questionable whether hospitalisation is necessary. Thus, in the absence of an agreed standard care for IV loop diuretics,^24^ we opted for 240 mg per day given as a continuous infusion.

IV loop diuretic dose can be titrated by the treating team if it is felt clinically necessary. If a patient reaches euvolaemia before completion of the trial, then they will be withdrawn from the study.

### Trial visits

**Figure 2:**
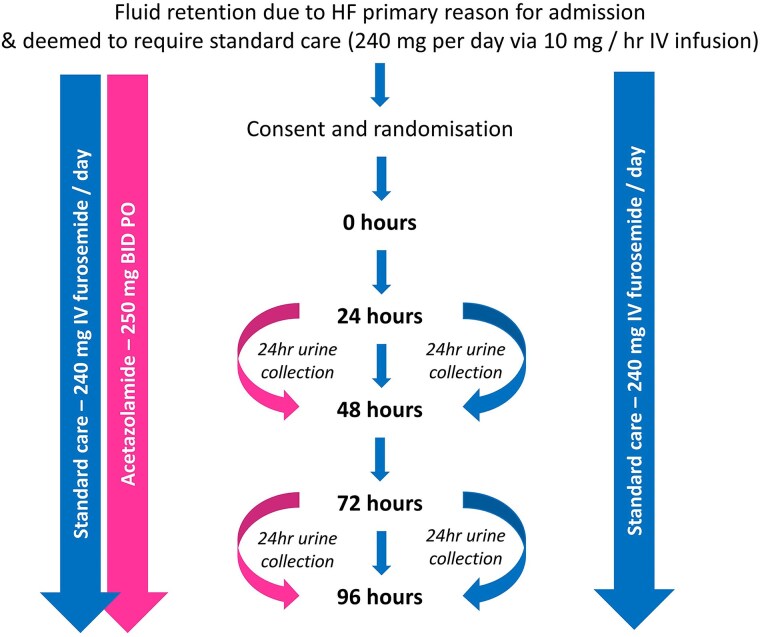
Study visits. HF, heart failure; IV, intravenous; IVC, inferior vena cava; hr, hour; BID, bis in dia; PO, per os.

The study team will visit patients on a daily basis for 5 days after randomisation (*Figure 2*). Medication will be prescribed electronically and administered by ward staff. Net fluid loss (fluid intake—urine output) will be calculated on days 2–5 by trial staff using routinely collected data on the ward. Vital signs and daily blood tests will be taken as part of routine care. Trial staff will measure the patient’s body weight and assess breathlessness at rest and on exertion using a 5-point Likert scale each day (1—not breathless; 5—extreme breathlessness).^25^

Twenty-four-hour urine collections will begin at 24 and 72 h after the first dose of acetazolamide. Patients will be asked to void their bladder and then begin the collection. Urinary catheterisation is optional. Inferior vena cava (IVC) diameter will be measured on day 1 and day 5 to assess changes in ultrasound measures of congestion.^26^

Patients will not have a fluid restriction due to the negative impact on quality of life,^27^ and the use of net fluid loss rather than urine output as the primary endpoint will negate any increased diuresis due to increased oral intake.

### Safety monitoring, sponsor and funding

This is an investigator-led trial. The primary investigator (C.J.J.) will have oversight of all trial activities, including safety monitoring and reporting. Trial monitoring will be performed by the sponsor—Hull University Teaching Hospitals NHS Trust Research and Development Department. ADA-HF is funded by the British Heart Foundation Clinical Research Collaborative Research Development Fund.

## Statistical plan

### Sample size calculations

#### Net fluid loss

We estimate that a clinically significant difference in daily net fluid loss is 500–1000 mL per day—a minimum of 4000 mL over an 8 day period (median length of hospitalisation in the UK) and 2 L over the 4 day period of our study. Such an increase in net fluid loss would indicate a significant increase in diuresis that may shorten hospital admission. The difference in urine output was 500 mL after 2 days treatment with acetazolamide in the ADVOR trial,^19^ and 375 mL after 1 day treatment with hydrochlorothiazide in the CLOROTIC trial.^28^

We expect an average daily fluid loss of 1000 mL per day with a standard deviation (SD) ranging from 500 to 1000 mL (*Table 2*). We anticipate a drop-out rate of 10% but given the lack of reliable clinical data on the tolerability of acetazolamide, we anticipate a higher drop-out rate of 20% in the acetazolamide arm. Thus, to detect a difference of 500–1000 mL in net fluid loss per day with statistical power of 80% and a two-tailed significance of 5%, we would need to recruit a minimum of 35 patients (17 in the control arm and 18 in the acetazolamide arm).

**Table 2:** Net fluid loss in trials of diuretics in patients admitted with HF

Trial	*n* (trial arm)	Loop diuretic dose per day (mean)	Time period over which net fluid loss was measured	Net fluid loss (mL)
DOSE	151 (low dose)	120 mg	72 h	3575 ± 2635
	157 (high dose)	260 mg	72 h	4899 ± 3479
TACTICS-HF	128 (placebo)	140 mg	24 h – Day 1	1541 ± 1525
			24 h – Day 2	1419 ± 1379
			24 h – Day 3	1401 ± 1387
ATHENA-HF^a^	178 (standard care)	120 mg	24 h	1183 (510–1955)
			48 h	2282 (1155–4135)
			72 h	3810 (2011–5565)
			96 h	5584 (2924–8132)
UNLOAD	100 (standard care)	180 mg	48 h	3300 ± 2610
CARESS-HF	94 (standard care)	NR	96 h	7082 ± 4183
ADVOR	259 (placebo)	120 mg	48 h	4100 ± 1800
CLOROTIC^a^	116 (placebo)	80 mg	24 h	1400 (1100–2162)

Results presented as mean ± SD apart from

^a^where results reported as median (interquartile range).

NR, not reported.

#### Serum chloride concentrations

Data on clinically significant changes in serum chloride levels are lacking. The normal range for chloride is 96–109 mmol/L. In observational studies,^6,7^ 85–90% had normal on admission. Of those with normal chloride on admission, 10–28% had developed hypochloraemia by discharge. Among those who developed hypochloraemia by discharge the median fall in chloride was 8 (5–12) mmol/L (*Table 3*). We cannot estimate the minimum clinically meaningful difference in chloride but patients who develop hypochloraemia during admission are at much greater risk of adverse outcome.^6,7^ The median duration of admission for patients admitted in the UK is 8 days, to detect a difference of ∼1 mmol/L per day with 80% power and 5% two-tailed significance with the above anticipated drop-out rate we would need to recruit a minimum of 14 patients in the control arm and 17 patients in the ACZ arm.

**Table 3:** changes in serum chloride concentrations during in-patient diuresis

Study	Design (*n*)	Daily IV diuretic dose in FE	LoS (days)	Hypochloraemia on or during admission—normal chloride concentration on discharge		Normal chloride on admission—hypochloraemia on discharge	
				Median ΔCl^−^ admission to discharge (mmol/L)	SD (mmol/L)	Median ΔCl^−^ admission to discharge (mmol/L)	SD (mmol/L)
Ter Maaten^6^	Retrospective cohort. PROTECT study. (*n* = 1960)	80 mg	14	5 (4–9)	NR	−7 (−10–5)^a^	NR
Cuthbert^7^	Retrospective cohort. Unselected in-patients with HF. (*n* = 1002)	NR	12	6 (5–8)	3	−8 (−12–−5)	3

^a^These data must be erroneous as patients who have developed hypochloraemia since admission cannot have increases in serum chloride levels; we included it as it is the only published data available from which we can estimate clinically meaningful changes in serum chloride levels.

Cl^−^, serum chloride concentrations; HF, heart failure; IV, intravenous; SD, standard deviation.

We will conduct both an intention-to-treat analysis and a per-protocol analysis in which only patients who completed the study without deviating from the protocol will be included. Thus, we aim to recruit a maximum of 50 patients. The extra patients recruited will also act as a contingency should any patient need to be removed from the analyses for any reason.

### Randomisation and blinding

Randomisation will be performed by a simple web-based system. Patients will be randomized 1:1 to either acetazolamide or standard care in a random permuted block of up to 50 patients. The trial is an open-label investigation as the primary endpoints and most secondary endpoints will not be susceptible to bias from unblinded treatment.

### Analysis

We will assess the distribution of daily and total fluid loss, and change in serum chloride concentrations in both arms. If there is normal distribution and equality of variance, we will compare the median net fluid loss using an independent samples *t*-test; if the distribution is not normal, we will use the Mann–Whitney U-test. A *P* value *≤*0.05 will indicate a statistically significant difference between the two arms.

Normality of distribution will be tested using distribution curves. Equality of variance will be tested using Levene’s test: the samples will be assumed to have equal variance if Levene’s test is *P* > 0.05. We do not expect any missing data, but any patient with missing data for each of the co-primary endpoints will be excluded from the analysis.

Secondary outcomes are exploratory, and none are powered for statistical significance. Secondary outcomes that are measured as continuous variables will be assessed for normality of distribution and equality of variance as described above, and differences will be assessed using independent samples *t*-test for normally distributed data, and the Mann–Whitney U-test for non-normally distributed data. Secondary outcomes that are measured as categorical will be assessed using χ^2^ tests, and un-adjusted logistic regression (with treatment assignment as the independent variable). Survival analysis (time to cause specific hospitalisation and time to cause specific mortality) will be conducted with a time-to-first event analysis using Kaplan-Meier and log-rank tests, unadjusted Cox proportional-hazard outcome models.

## Discussion

IV loop diuretics as the standard of care for patients admitted with fluid retention due to HF have not changed for more than half a century.^4^ Combination diuretic therapy may increase diuresis, shorten hospital stay, reduce the time spent with disabling symptoms, and, potentially, improve outcome.^29^ Acetazolamide is one of many possible ‘adjunctive’ diuretic treatments, but more data as to the safety and efficacy of acetazolamide is needed. Despite the publicity surrounding it, ADVOR has limited applicability, and ADA-HF will offer complementary data.^30^

### ADA-HF and ADVOR

The dose of IV loop diuretic used as the comparator to combination diuretic treatment with acetazolamide in ADVOR was low (120 mg furosemide equivalents per day).^17^ This is was similar to the median dose of IV loop diuretic in the low-dose arm of the DOSE trial, which was inferior to high-dose treatment (2.5 × usual dose, mean ∼250 mg per day) for diuresis, and only a fraction of what was permitted in the pharmacological arm of the CARESS trial.^23,31^

The benefit of acetazolamide in ADVOR was entirely driven by patients receiving <120 mg of furosemide equivalents per day [hazard ratio (HR) for the primary endpoint of decongestion by 48 h in patients receiving ≤120 mg IV furosemide per day = 1.78 [95% confidence interval (CI) 1.33–2.36] compared with 1.08 (95% CI 0.76–1.55) in patients receiving >120 mg per day].^19^

Only a third of patients in ADVOR initially had oedema above the knee. In the UK, many patients with mild oedema (below knee) are managed without hospitalisation,^1^ particularly if the oral dose of loop diuretic is low, as was the case in ADVOR (median 60 mg per day prior to admission).^19^

Most adjunctive diuretics, such as thiazide diuretics and tolvaptan, are given orally, and IV acetazolamide may be difficult to obtain in some hospitals. Whether oral acetazolamide has a similar diuretic effect to IV acetazolamide (as used in ADVOR) is unknown. Oral acetazolamide at doses between 250 and 750 mg daily may increase diuresis when used in combination with loop diuretics or spironolactone.^32^ However, none of these studies used high-dose loop diuretic treatment as the comparator.

Finally, acetazolamide was only given for 48 h in the ADVOR trial, and although it was not associated with a greater rate of adverse events than IV loop diuretic alone, it is likely that in practice acetazolamide will be given for longer periods. The safety of acetazolamide given over longer periods in patients admitted to the hospital with HF is unknown. Early trials found that side effects such as drowsiness and paraesthesia are common (14–38%) at doses >1000 mg per day, and less common (but not infrequent) at doses ≤500 mg per day (5–10%).^33,34^

### ADA-HF and hypochloraemia

Hypochloraemia is an important marker of a poor prognosis in patients with HF, but whether it *causes* a poor prognosis is unclear.^8^ Observational data suggest that patients with persistent hypochloraemia (hypochloraemia on admission and discharge) or incident hypochloraemia (normal chloride concentration on admission but hypochloraemia on discharge) have a worse prognosis than patients with normal serum chloride concentrations throughout admission.^6,7^

There are mechanisms by which hypochloraemia may drive worsening HF and diuretic resistance,^8^ and prevention or correction of hypochloraemia may improve prognosis. Acetazolamide may increase serum chloride levels during treatment with other diuretics in patients with HF, but this has not been tested in an RCT.

### Feasibility outcomes

Combination diuretic therapy has many possible advantages, but without robust, generalizable data, it may be under-used. Although ADVOR and CLOROTIC are important first steps, acetazolamide and hydrochlorothiazide are only two of multiple possible adjunctive diuretic treatments that need to be investigated.^4^ Whether large-scale RCTs of diuretic interventions are feasible in patients with HF admitted to hospitals in the UK is unknown. Data from the ADA-HF trial will inform and influence recruitment strategies for future planned trials of diuretic strategies.

### Trial status

Protocol version 3 (date); recruitment began on 14 March 2023 and ended on 31 January 2024.

## Conclusion

ADA-HF will offer complementary data to the ADVOR study regarding the safety and efficacy of oral acetazolamide as a combination diuretic treatment alongside high-dose IV furosemide for patients admitted to hospital with severe fluid retention due to HF. It will also demonstrate whether acetazolamide acts as a chloride-sparing diuretic: preventing or correcting hypochloraemia may improve prognosis for patients admitted with HF. Finally, ADA-HF will provide valuable information on the feasibility of conducting large-scale diuretic trials in patients admitted to hospital with HF in the UK.

## Data Availability

Data will be made available after reasonable request to the chief investigator.

## Lead author biography

Dr Joe Cuthbert BSc (hons) MBBS MD FHEA, Senior Lecturer in Cardiology, Hull York Medical School, University of Hull, Honorary Consultant Cardiologist, Hull University Teaching Hospitals NHS Trust Dr Joe Cuthbert is a Senior Lecturer in Cardiology at the Hull York Medical School. He has established himself as an up-and-coming HF researcher with expertize in diuretics and treatment of congestion with multiple publications in high-impact peer-reviewed journals. He was awarded a grant from the British Heart Foundation for the ADA-HF trial of acetazolamide in patients admitted to hospital with HF and treated with high dose loop diuretic. The results will provide the foundations of further work in the field of HF and diuretic treatment in the UK.
